# *Notes from the Field*: Outbreak of *Vibrio cholerae* Associated with Attending a Funeral — Chegutu District, Zimbabwe, 2018

**DOI:** 10.15585/mmwr.mm6719a6

**Published:** 2018-05-18

**Authors:** Jarred B. McAteer, Sydney Danda, Tonderai Nhende, Paul Manamike, Tonderai Parayiwa, Andrew Tarupihwa, Ottias Tapfumanei, Portia Manangazira, Gibson Mhlanga, Daniela B. Garone, Andrea Martinsen, Rachael D. Aubert, William Davis, Rupa Narra, Shirish Balachandra, Beth A. Tippett Barr, Eric Mintz

**Affiliations:** ^1^Epidemic Intelligence Service, CDC; ^2^Division of Foodborne, Waterborne, and Environmental Diseases, National Center for Emerging and Zoonotic Infectious Diseases, CDC; ^3^Chegutu District Hospital; ^4^Chegutu Municipality; ^5^National Microbiological Reference Laboratory; ^6^Ministry of Health and Child Care, Zimbabwe; ^7^Médecins Sans Frontières, Zimbabwe; ^8^Emergency Response and Recovery Branch, Division of Global Health Protection, Center for Global Health, CDC; ^9^CDC-Zimbabwe, Harare.

On January 16, 2018, the Zimbabwe Ministry of Health and Child Care (MoHCC) was notified of five adults with watery diarrhea and severe dehydration who were admitted to Chegutu District Hospital, Mashonaland West Province. Three of the five patients died within hours of admission. *Vibrio cholerae* O1 serotype Ogawa was isolated from the stool sample of one decedent, prompting an investigation. During 2008–2009, Zimbabwe experienced one of the largest and deadliest cholera outbreaks in recent history (98,585 cases and 4,287 [4.3%] deaths), during which Chegutu reported a case fatality rate (CFR) >5% (*1*,*2*). During 2012–2016, Zimbabwe reported 93 cholera cases and two deaths nationwide, but the increasing population density and aging water and sanitation infrastructure in Chegutu raised concern about the possibility of another widespread outbreak.

MoHCC identified the index patient as a woman aged 79 years who died on January 8 after 2 days of watery diarrhea. Before her death, she sought care at a private clinic, but cholera was not suspected at the time. In accordance with local practice, water was flushed through the woman’s body to cleanse it in preparation for burial; the water was subsequently discarded into the municipal sewer network without further treatment. One person who had been involved in preparation of the body and who served traditional food at the multiday funeral reception at the index patient’s home developed watery diarrhea 2 days after the funeral. Six other funeral attendees, including all three decedents, had reported developing acute watery diarrhea within 6 days of the funeral. Two of the patients who subsequently died had reported assisting with the burial.

Within 4 days of the index patient’s funeral, the outbreak had spread to local residents who reported no epidemiologic links to the funeral (Figure). During this time, intermittent interruptions of the chlorinated municipal water supply and low pressure areas might have increased the use of unchlorinated boreholes and shallow wells that are vulnerable to contamination from adjacent, poorly maintained, sewer pipes, including those containing water used to wash the body. Microbiologic testing from a shallow well at the funeral reception location yielded fecal coliform bacteria, suggesting conditions conducive to cholera transmission.

**FIGURE:**
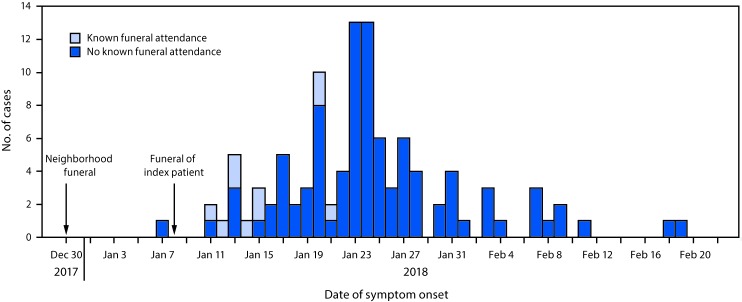
Number of reported cholera cases, by known date of symptom onset (N = 106*) and the patient’s funeral attendance status — Zimbabwe, December 30, 2017–April 5, 2018 * Date of symptom onset was unknown for one additional patient.

Although the index patient had not reported any travel, the epidemiologic investigation revealed that her home was less than a half mile from the site of a separate funeral that had taken place on December 30, 2017. Although that death was not associated with a diarrheal illness, two persons who attended the funeral had traveled 292 miles (470 km) from Lusaka, Zambia, where a cholera outbreak was ongoing (*3*). These attendees did not report diarrhea and were not tested for asymptomatic carriage of *V. cholerae*. It is not known how the index patient became infected; however, it is likely that funeral practices employed to prepare her body for burial and unsafe food preparation at the subsequent funeral potentiated the wider geographic distribution of this outbreak.

Following a coordinated rapid response effort including surveillance, health promotion, laboratory testing, case management training, and emergency water, sanitation, and hygiene (WASH) activities by MoHCC and international partners, the Zimbabwe outbreak was contained to urban and peri-urban areas of Chegutu. A single suspected case was identified along the major highway from Chegutu to the capital of Harare, 62 miles (100 km) away, but no cases were identified in Harare.

As of April 5, 2018, a total of 107 cases, including 51 hospitalizations and four deaths (CFR = 3.8%) had been reported in Zimbabwe; 9% of the cases occurred in children aged <5 years. The last case was reported on February 19. Approximately 60% of the cases occurred in three suburbs: Chegutu township (19; 17%), Pfupajena (31; 29%), and Kaguvi (13; 12%). Of 64 stool specimens tested from January 10 to February 21, nine (14.1%) yielded *V. cholerae* O1. Antimicrobial susceptibility testing by disk diffusion identified eight isolates with decreased susceptibility to cotrimoxazole, and two with decreased susceptibility to tetracycline. All isolates were susceptible to ciprofloxacin, considered first line therapy for severe cholera in accordance with Zimbabwe national guidelines.

In the setting of cholera epidemics, localized outbreaks have been associated with funeral gatherings, including transporting and washing or preparing a body for burial and contamination of shared meals at a funeral (*4*,*5*). However, outside of epidemics, cholera outbreaks rarely originate from funeral gatherings (*6*). Given the increase in regional travel to and from countries experiencing cholera outbreaks and those with endemic cholera transmission (*7*), the potential for cholera outbreaks should be considered an ever-present risk in areas that lack adequate WASH infrastructure. Early detection and promotion of safe handling of the dead are part of the routine recommendations during a cholera outbreak. This outbreak is a reminder that even in settings where cholera has been absent, public messaging about safe burial and safe food handling need to be provided at all times.

## References

[1] Mukandavire Z, Liao S, Wang J, Gaff H, Smith DL, Morris JG Jr. Estimating the reproductive numbers for the 2008-2009 cholera outbreaks in Zimbabwe. Proc Natl Acad Sci U S A 2011;108:8767–72. 10.1073/pnas.1019712108 21518855 PMC3102413

[2] World Health Organization. Daily cholera update and alerts. July 30, 2009. Geneva, Switzerland: World Health Organization; 2009. http://www.who.int/hac/crises/zwe/sitreps/zimbabwe_cholera_update_30july2009.pdf?ua=1

[3] Sinyange N, Brunkard JM, Kapata N, Cholera epidemic—Lusaka, Zambia, October 2017–May 2018. Morb Mortal Wkly Rep 2018,67:556–9. 10.15585/mmwr.mm6719a5 PMC6048949 29771877

[4] Acosta CJ, Galindo CM, Kimario J, Cholera outbreak in southern Tanzania: risk factors and patterns of transmission. Emerg Infect Dis 2001;7(Suppl):583–7 . 10.3201/eid0707.017741 11485679 PMC2631835

[5] Gunnlaugsson G, Einarsdóttir J, Angulo FJ, Mentambanar SA, Passa A, Tauxe RV. Funerals during the 1994 cholera epidemic in Guinea-Bissau, West Africa: the need for disinfection of bodies of persons dying of cholera. Epidemiol Infect 1998;120:7–15. 10.1017/S0950268897008170 9528812 PMC2809343

[6] Korthuis PT, Jones TR, Lesmana M, An outbreak of El Tor cholera associated with a tribal funeral in Irian Jaya, Indonesia. Southeast Asian J Trop Med Public Health 1998;29:550–4. 10437955

[7] Ali M, Nelson AR, Lopez AL, Sack DA. Updated global burden of cholera in endemic countries. PLoS Negl Trop Dis 2015;9:e0003832 . 10.1371/journal.pntd.0003832 26043000 PMC4455997

